# Proteolytic Landscapes in Gastric Pathology and Cancerogenesis

**DOI:** 10.3390/ijms23052419

**Published:** 2022-02-22

**Authors:** Sabine Bernegger, Miroslaw Jarzab, Silja Wessler, Gernot Posselt

**Affiliations:** 1 Division of Microbiology, Department of Biosciences and Medical Biology, Paris Lodron University of Salzburg, Hellbrunner Strasse 34, 5020 Salzburg, Austria; sabine.bernegger@plus.ac.at (S.B.); miroslaw.jarzab@plus.ac.at (M.J.); silja.wessler@plus.ac.at (S.W.); 2 Cancer Cluster Salzburg and Allergy Cancer BioNano Research Centre, University of Salzburg, Hellbrunner Strasse 34, 5020 Salzburg, Austria

**Keywords:** gastric cancer, protease, MMP, ADAM, HtrA, EMT, *Helicobacter pylori*, E-cadherin

## Abstract

Gastric cancer is a leading cause of cancer-related death, and a large proportion of cases are inseparably linked to infections with the bacterial pathogen and type I carcinogen *Helicobacter pylori*. The development of gastric cancer follows a cascade of transformative tissue events in an inflammatory environment. Proteases of host origin as well as *H. pylori*-derived proteases contribute to disease progression at every stage, from chronic gastritis to gastric cancer. In the present article, we discuss the importance of (metallo-)proteases in colonization, epithelial inflammation, and barrier disruption in tissue transformation, deregulation of cell proliferation and cell death, as well as tumor metastasis and neoangiogenesis. Proteases of the matrix metalloproteinase (MMP) and a disintegrin and metalloproteinase domain-containing protein (ADAM) families, caspases, calpain, and the *H. pylori* proteases HtrA, Hp1012, and Hp0169 cleave substrates including extracellular matrix molecules, chemokines, and cytokines, as well as their cognate receptors, and thus shape the pathogenic microenvironment. This review aims to summarize the current understanding of how proteases contribute to disease progression in the gastric compartment.

## 1. Introduction

Cancer is a leading cause of premature death in 127 countries, and if current numbers and trends continue, cancer could overtake cardiovascular disease in this century [1]. Among all common cancer types, stomach cancer accounted for 5.6% of all new cancer cases and 7.7% of cancer-related deaths in 2020 [2]. Gastric cancer appears as two pathological variants. The diffuse type is characterized by the development of linitis plastica and is associated with an unfavorable prognosis due to a heritable loss-of-function mutation of the E-cadherin gene CDH1. The intestinal type of gastric cancer is mainly considered as an infectious disease, since persistent colonization with the human pathogen *Helicobacter pylori* (*H. pylori*) has been discovered as the main cause. Although erosive gastritis can be caused by stress, alcohol, or chemical drugs, up to 89% of non-cardia gastric cancers are attributable to *H. pylori* infection [3]. Several infections are associated with cancer development, however, it was estimated that out of 2.2 million infection-attributed cancer cases diagnosed worldwide, *H. pylori* was the primary cause of 810,000 cases in 2018 [4]. 

A major problem in gastric cancer therapy is late diagnosis, as the early stages of gastric cancer are usually clinically asymptomatic. *H. pylori*-mediated, inflammation-driven gastric cancer development is a multistep process taking decades and is described as the “Correa cascade”. It is characterized by a prolonged precancerous process with well-defined sequential stages initiated by chronic active gastritis, chronic atrophic gastritis, intestinal metaplasia, and dysplasia, which can finally result in invasive carcinoma [5,6]. Intensive research has revealed a large set of different pathogenic factors originated from *H. pylori* that induce a complex network of molecular and cellular mechanisms leading to *H. pylori*-mediated inflammatory responses, and subsequently, carcinogenesis, which have been summarized recently in excellent review articles [7,8,9,10].

Awareness of the importance of proteases in gastric cancer has increased in recent years. Different types of proteases, including a number of (matrix) metalloproteases, serine proteases, collagenases, and so on, derived from either the epithelium, the immune cell infiltrate, or *H. pylori* are involved in numerous pathogenesis-associated signaling pathways. They contribute to the release of signaling molecules, cleavage of cell surface proteins, and modulation of the extracellular matrix to ensure successful colonization of the gastric epithelium and foster subsequent pathologic events [11,12]. These processes involve the immediate early pro-inflammatory response, the loss of the epithelial integrity, or the induction of the epithelial-mesenchymal transition (EMT) and cancerogenesis in the polarized epithelium.

## 2. The Role of Proteases in *H. pylori* Colonization and Mucosal Inflammation

A high percentage of gastric adenocarcinomas (GACs) are linked to *H. pylori* infection or autoimmune gastritis, and thus GAC is considered a paradigm of inflammation-driven carcinogenesis [13,14,15]. Although the pathogenicity of intestinal-type and diffuse-type carcinoma differs also in their correlation with *H. pylori* colonization [16], both are associated with genetic predispositions in a number of inflammatory mediators [17]. 

**Figure 1:**
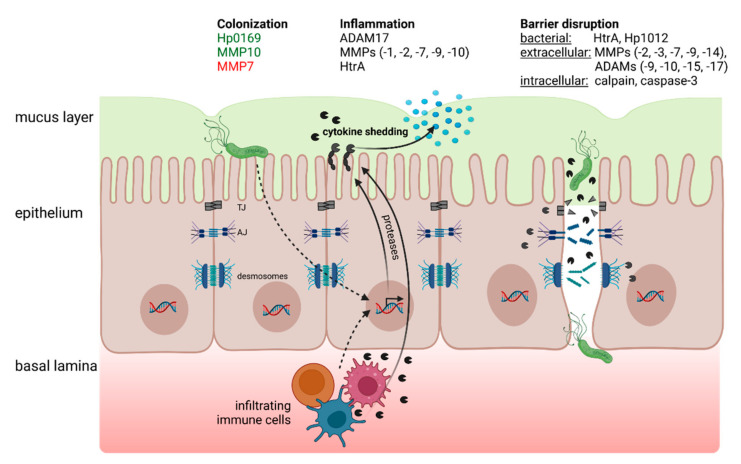
Proteases in colonization, epithelial inflammation, and epithelial barrier disruption. Colonization of the gastric tissue by *H. pylori* is positively (green) or negatively (red) affected by the activity of several proteases of human or bacterial origin. Additionally, infection with *H. pylori* leads to upregulation of proteolytic activities through elevated transcription levels of proteases in epithelial cells, or through the enhanced immune cell infiltrate. These proteases from epithelial, immune cell, or bacterial origin are directly involved in promoting mucosal inflammation and disruption of the gastric epithelial barrier through their involvement in cytokine shedding, degradation of ECM proteins, and opening of lateral cell–cell junctions.TJ, tight junctions; AJ, adherens junctions. Created with BioRender.com.

The importance of bacterial and host proteases for a successful epithelial colonization by *H. pylori* has been investigated in a number of studies. The epithelial lining of the stomach is covered by a thick mucus layer, which facilitates transport of chyme and provides protection of the stomach tissue against chemicals and pathogens. Despite initial studies reporting the existence of an unknown *H. pylori* protease that softens mucus and supports colonization, no definite mucinase has yet been identified in *H. pylori* [18,19]. However, motility through the dense mucus layer is accomplished by altering the viscoelastic properties via pH and urease-dependent mechanisms [20]. Amongst *H. pylori* proteases, the expression and secretion of the collagenase Hp0169 was shown to be a prerequisite for bacterial colonization in vivo [21]. Hp0169 was described to act as a true collagenase able to degrade native triple-helical type I collagen in the ECM and facilitate bacterial adherence [21]. A significant role of the collagen and extracellular matrix in bacterial colonization is supported by the fact that host proteases of the matrix-metalloproteinase (MMP) family targeting collagen, such as MMP7 and MMP10, also affect *H. pylori* colonization levels (Figure 1). A number of MMPs are upregulated and activated during *H. pylori* gastritis and gastric cancer (cf. Section 4 and Section 5). Interestingly, MMP10 supports colonization, whereas MMP7 levels are inversely correlated with bacterial burden [22,23]. The opposite effect of these host proteases could be linked to their association with the host immune response and inflammation, rather than to their direct proteolytic activity in the tissue. Levels of infiltrating B cells and T cells, and in particular Th1- and Th17-associated cytokines like interferon gamma (Ifn-γ) and interleukin-17 (IL-17), are elevated in MMP7 knockout mice [24]. On the other hand, in concert with IL-22, MMP10 fosters CD8+ T-cell-mediated tissue inflammation, which aids *H. pylori* survival, and deletion of MMP10 is associated with reduced tissue colonization [23].

In particular, ECM-targeting proteases are important determinants shaping the micromilieu and support the establishment of persistent *H. pylori* infections in a controlled pro-inflammatory environment. Epithelial colonization with *H. pylori* has a strong impact on the local tissue microenvironment [25]. In addition to the local inflammatory response in the epithelium, epithelial mediators attract a massive immune cells infiltrate. Immigrating neutrophils, macrophages, and lymphocytes drastically change the cellular composition of the gastric mucosa [26,27]. In many cases, the literature does not identify the origin of the individual factors that form the microenvironment, and it remains unclear whether the factors are produced by the inflamed tissue, the newly recruited immune cell infiltrate, or both. Despite particular ambiguities in the producing source, a clear increase in proteolytically active (metallo-)protease levels is seen in the inflammatory microenvironment in the gastric mucosa (Table 1). A disintegrin and metalloproteinase domain-containing protein 10 (ADAM10), -17, and -19 are upregulated in *H. pylori*-infected individuals, and ADAM9, -12, -15, and -20 are deregulated in cancer biopsies [28,29,30]. Similarly, a variety of MMPs are upregulated in vivo in *H. pylori* gastritis (MMP1, -8, -9, and -10) and in malignantly transformed tissue (MMP2, -7, -9, -11, -12, -14) [31,32,33,34,35,36,37]. In vitro infection experiments with gastric cancer cell lines support a direct regulation of MMP1, -3, -7, -8, -9, and -10 in the epithelium in response to *H. pylori* infection [34,38,39]. The impact of the proteolytic activities in the inflamed mucosa on the inflammatory tissue microenvironment is twofold. First, direct effects on inflammatory mediators and cytokines are seen. ProTNF-α is canonically processed and shed from producing cells in an ADAM17-dependent manner [40,41], but MMP1 and MMP7 have also been suggested as potential TNF-α sheddases [42,43]. ADAM17 also contributes to proTGF-α processing in *H. pylori* infections [44], whereas MMP1 is involved in cleaving proIL-1β [42]. Other studies indicate that MMP7 dampens the production of the cytokines IL-1β, MIP-1β, TNF-α, IP-10, RANTES, and IL-17, as MMP7-deficient mice produced higher levels as compared to wild-type animals [22]. This is in line with studies reporting overshooting inflammation in MMP7 knockout animals and thus suggest a protective role of MMP7 in vivo [24]. Not only are cytokines affected by proteolytic processing, but the chemoattractant potency of chemokines is also subject to modification by targeted cleavage events. It has been demonstrated that CXCL5 and CXCL8 potency can be amplified in a gelatinase (i.e., MMP2 and -9)-dependent manner and results in boosted neutrophil recruiting, whilst other chemokines such as CXCL1 are inactivated by cleavage [45,46]. A pronounced effect of the MMP10-CXCL16 axis was also observed on the recruitment of a CD8+ T cell infiltrate [23]. Therefore, the proteolytic constituents in the tissue microenvironment are influencing the inflammatory and chemo-attractive properties at the site of inflammation [47]. In return, the resulting tissue microenvironment governs protease production. For instance, the gelatinases MMP2 and -9 are upregulated in a Th17 environment via IL-21 [48,49] and tissue levels of IL-1β are closely linked to MMP3 expression in vivo [50]. MMP7 production depends on gastrin [51], which is considered an important mediator of gastric tumorigenesis [52]. In addition to cytokines and chemokines, growth factor activity also is subject to regulation via protease-dependent mechanisms. HB-EGF (heparin-binding EGF-like growth factor) shedding is observed in response to ADAM17 activation [53], and MMP7 was also suggested as a HB-EGF sheddase [54]. HB-EGF as well as TGF-α activity results in epidermal growth factor receptor signaling, which is associated with a local stimulation of cell proliferation [44,55]. Second, proteases target the extracellular matrix in the inflamed tissue and thus aid the recruitment of an immune cell infiltrate, which supports inflammation and contributes to pathogenic tissue remodeling. Most prominently, the gelatinase MMP9 is correlated with tissue-infiltrating macrophages [56]. The concerted action of proteases derived from the epithelium, infiltrating and activated immune cells, as well as *H. pylori* proteases foster disease progression. MMP-dependent ECM remodeling is not only linked to inflammation but also directly contributes to gastric ulceration [57,58]. Besides the abovementioned host proteases, the *H. pylori* protease high temperature requirement A (HtrA) was suggested to contribute to chronic inflammation, as the leucine 171 variant of HtrA was associated with higher inflammation scores and elevated serum gastrin levels [59]. The authors speculate that altered HtrA activity in the S171L HtrA variant could potentially mediate more efficient migration over the epithelial barrier and thus influence inflammation and gastrin production [59]. HtrA was originally discovered as a periplasmic serine protease and chaperone with essential functions in *H. pylori* growth and survival [60,61,62]. As a secreted protease, HtrA exerts important functions in the disintegration of the gastric epithelium via targeting epithelial junctions (cf. Section 3).

Acute *H. pylori* infection induces hypochlorhydria, which facilitates the colonization by the bacteria. In chronic infections, corpus-predominant colonization and pan-gastritis results in acid hypo-secretion, whilst antrum-predominant gastritis is often associated with acid hyper-secretion [87,88]. The reduction in acid production in *H. pylori* infections was at least partly attributed to an ADAM17-dependent transcriptional repression of the gastric H, K–adenosine triphosphatase α-subunit [63].

## 3. Proteolytic Impairment of Junctional Integrity and Epithelial Barrier Function

The gastric mucosal epithelium represents one of the main contact areas with the outside world and provides an effective protective barrier, which is characterized by a high order of 3D organization and cellular polarization. It features numerous glands and pits and a defined distribution of the various cell types specialized in fulfilling these complex requirements. Besides mucus-secreting cells, specialized cells such as parietal cells, chief cells, and entero-endocrine cells mediate the production of gastric acid, the production and secretion of digestive and antimicrobial enzymes, and hormones, respectively [89,90,91]. A prerequisite for the integrity and maintenance of the gastric epithelial barrier is the maintenance of cell polarity facilitated by several adhesive cell–cell connections, including tight junctions (TJ), adherens junctions (AJ), and desmosomes [92] (Figure 1). Structurally, these junctions consist of transmembrane proteins as central key molecules that mediate the intercellular adhesion and form intracellular protein complexes to stabilize the junctions and link them to the actin cytoskeleton. They control several important signal transduction pathways involved in inflammation and carcinogenesis [93,94,95,96,97].

In healthy individuals, the maintenance of a functional epithelial barrier requires a continuous cell turnover accompanied by homeostatic changes in intercellular junction proteins [98]. In recent years, it became evident that proteolytic impairment of barrier functions is strongly linked to the disease progression in gastric cancer pathogenesis. This has been demonstrated for deregulated host proteases and proteases expressed by pathogenic bacteria such as *H. pylori*.

TJs are crucial for maintaining apical-basolateral cell polarity and are responsible for the regulation of paracellular permeability. TJs are a network of proteins at the lateral cell surface of epithelial cells, including claudins, occludin, the junctional adhesion molecule-A (JAM-A), and intracellular scaffold proteins, such as zonula occludens (ZO) and tricellulin [99]. Recently, it has been shown that the four-span transmembrane TJ proteins occludin and claudin-8 are cleaved in an extracellular loop during infection of gastric epithelial cells with *H. pylori*. Cleavage of the two TJ proteins was attributed to the secreted bacterial serine protease HtrA [81]. Cleavage of occludin and members of the claudin protein family can also occur through upregulation of host proteases, such as MMP2, MMP7, and MMP9, and is usually associated with an increase in epithelial or endothelial permeability [70,74,100,101,102,103]. Although these proteases were shown to be induced in response to *H. pylori* infection [39,65] and in gastric cancer tissue [104,105], a direct role of these proteases in disruption of the gastric epithelium has not been established so far. Recently, JAM-A has been described as a target of *H. pylori*, and Hp1012 was suggested as the responsible protease. Although JAM-A is cleaved in response to *H. pylori* infection and by a protein fraction also containing Hp1012, direct cleavage of JAM-A by the recombinant protein was not shown. Nevertheless, the observed cleavage event in the intracellular JAM-A c-terminus results in impaired barrier function, reduced intercellular adhesion, and increased invasive potential of epithelial cells [78].

Beneath the TJs, AJs are located, in which the tumor suppressor E-cadherin represents the adhesive core component. E-cadherin is a glycosylated transmembrane protein with five extracellular cadherin-motifs, a single-pass transmembrane segment, and a short conserved cytoplasmic domain, which interacts with β-catenin, plakoglobin (γ-catenin), and p120-catenin. β-catenin also interacts with α-catenin that is linked to filamentous actin. β-catenin is a proto-oncogene as it plays an important role in the Wnt signaling pathway. Both β-catenin and p120-catenin exhibit a second role in the nucleus, where they control transcription factors like Tcf/lef and Kaiso, which regulate the expression of cancer-associated target genes, such as *c-myc*, *cyclin D1*, *mmp7*, and so on [106,107]. In normal epithelial cells, E-cadherin is constantly shed at a low rate from the cell surface, in the process of dynamic control of intercellular adhesions. However, aberrant ectodomain shedding of E-cadherin was reported in various types of cancer and has been suggested as a prognostic marker in gastric cancer [108,109]. Numerous soluble and membrane-anchored host proteases have been associated with E-cadherin shedding, such as the matrix metalloproteases MMP3, MMP7, MMP9, MMP14 [71,73,75,77], ADAM10, and ADAM15 [68,110], which are upregulated in response to *H. pylori* infection [38,39,65,66,111] and in gastric cancer tissue [104,105]. In addition to the induction of host proteases, *H. pylori* also exerts direct effects on E-cadherin shedding mediated by the HtrA protease that cleaves E-cadherin on infected gastric epithelial cells [79]. Apart from ectodomain shedding of E-cadherin, intracellular cleavage events in E-cadherin were also shown to severely impair epithelial integrity. In the context of *H. pylori* infections, upregulation of calpain and caspase-3 induced intracellular E-cadherin cleavage, resulting in disintegration of the E-cadherin/catenin complex and increased apoptosis of gastric epithelial cells [82,83,85].

Desmosomes represent the third major intercellular adhesion complex, and are located beneath AJ in the polarized epithelium. In the gastric mucosa, the desmosomal cadherins desmoglein-2 and desmocollin-2 represent the adhesive core components of desmosomes [112]. Although downregulation of desmoglein-2 has been associated with gastric cancer [113], not much is known about abnormal proteolytic cleavage of desmosomal cadherins in the context of gastric cancer progression. Recently, *H. pylori* HtrA was shown to directly cleave desmoglein-2 in the extracellular domain on gastric epithelial cells, thereby inducing a soluble desmoglein-2 fragment [80]. A similar fragment was associated with a compromised mucosal barrier function linked to matrix metalloproteases in settings of intestinal inflammation [114,115]. Moreover, enhanced desmoglein-2 shedding was associated with induction of MMPs, as well as ADAM9, ADAM15, and ADAM17, and impaired cell adhesiveness in squamous cell carcinoma [69,76]. Although no direct experimental evidence proves the impact of these proteases on desmosomes in gastric cancer, similar mechanisms appear plausible due to the documented expression of these proteases in response to *H. pylori* infection [30,116] and in gastric cancer [104,117]. 

Like the targeting of E-cadherin, intracellular cleavage of desmoglein-2 by caspases or calpain has been associated with induction of apoptosis in the inflamed intestinal epithelium [84,86]. Again, despite the lack of experimental data, it might be speculated that increased activation of caspase-3 and calpain by *H. pylori* could enhance intracellular desmoglein-2 cleavage and thus stimulate apoptosis in gastric epithelial cells.

Cleavage of TJ proteins, E-cadherin, and desmosome proteins locally opens intercellular adhesions and thus allows transmigration of *H. pylori* to the basolateral and basal domains of the polarized gastric epithelium [79,81]. Overcoming the epithelial barrier and gaining access to basolateral integrin-β1 for the delivery of the bacterial oncoprotein cytotoxin-associated gene A (CagA) is an integral disease mechanism in *H. pylori*-dependent carcinogenesis [118]. Although ADAM10 is activated in *H. pylori*-infected cells and cleaves E-cadherin [66], HtrA is the main E-cadherin protease in *H. pylori* infections [79,80]. 

Taken together, abnormal cleavage of junction proteins severely impairs the barrier properties of the gastric epithelium. On the one hand, reduction of functional cell–cell junction complexes reduces intercellular adhesion, which is usually associated with increased aggressiveness and invasiveness of carcinoma as it increases the migration and invasive potential of epithelial cells [119,120]. In particular, loss of E-cadherin is a characteristic step in epithelial-mesenchymal transition and, in the context of tumor progression, often plays a causative role in malignant transformation [121] (cf. Section 5).

## 4. The Role of Proteases in Proliferation, Cell Survival, and Neoplastic Transformation

**Figure 2:**
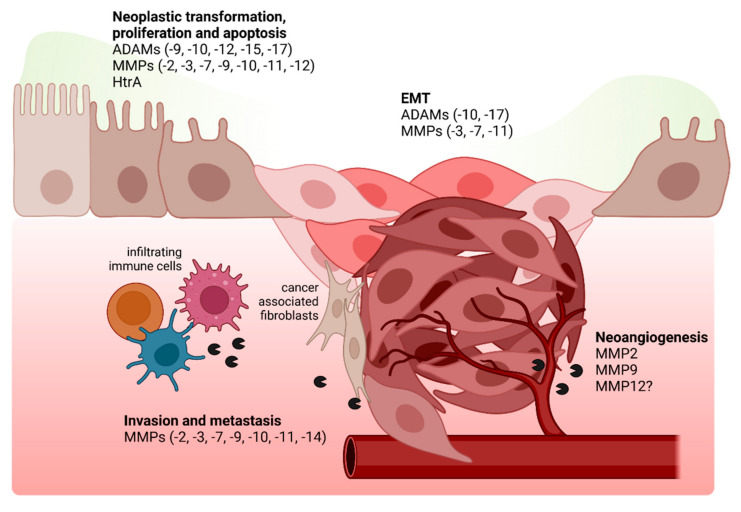
Proteases in EMT, tissue transformation, metastasis, and neoangiogenesis. EMT processes and the balance between cell survival and proliferation is influenced by a number of host and *H. pylori* proteases. At later stages of gastric cancer progression, proteases originating from *H. pylori* and epithelial cells in concert with proteases derived from tumor-associated immune cells or fibroblasts are directly involved in neoplastic transformation, as well as metastasis and neoangiogenesis. Created with BioRender.com.

Proteolytic activities feed into proliferative and anti-apoptotic signaling pathways and influence cell differentiation [122], and causal links between proteases and tumorigenic cell transformation have even been drawn (Figure 2) [123]. However, the mutational burden in gastric cancer does not point to specific protease-activated processes [124]. Nevertheless, a tumor-promoting role for ADAM and MMP proteins is clearly established in many cancer types, and their expression levels could serve as a prognostic marker in cancer (Table 2) [125]. In gastric cancer, MMP2, -3, -7, -9, -10, and -11 are upregulated in the course of disease progression, of which MMP2, -3, -7, and -9 have been suggested to have prognostic value for disease outcome [31,36,59,126,127,128,129,130,131]. MMP7 was shown to promote proliferation in non-transformed epithelial cells [132]. A meta-analysis revealed ADAM17 as a significant biomarker for poor prognosis in gastric cancer [133]. In contrast, beneficial effects of MMP12 expression have been discussed and expression levels were suggested to correlate inversely with disease outcome [37,134]. MMP11 levels also correlate with IGF-1 expression and IGF-1-stimulated proliferation [36,42]. Antibody-mediated inhibition of ADAM9 and -15 reduced in vitro proliferation of gastric cancer cell lines, whereas anti-ADAM12-treated cells produced higher proliferation rates [117]. ADAM17 induces pro-survival signaling via the EGFR signaling axis and protects from *H. pylori*-induced apoptosis. In this context, EGFR kinase inhibitors could extenuate premalignant pathology in gerbil models [44,135,136]. Additionally, the shedding and release of TNF-α via ADAM17 and several MMPs changes the balance between cell survival and apoptosis. [137]. Nevertheless the interrelation of MMPs and cell survival is ambiguous, and the same MMPs can exhibit both pro-apoptotic and anti-apoptotic activity [138].

Anti-apoptotic function of MMPs can be executed by cleaving the Fas ligand, shedding of tumor associated MHC complex class I-related protein, or activation of AKT/Protein kinase B. The pro-apoptotic activity of MMPs is often connected to changes in ECM composition and cleavage of adhesion molecules [64]. *H. pylori* infection itself also interferes with pro- and anti-apoptotic pathways. For instance, *H. pylori* TieA protein induced gastric epithelial cell death via Fas- and caspase-8-mediated apoptosis [143]. Nevertheless, *H. pylori* utilizes numerous strategies to reduce caspase-3-dependent apoptosis in infected host cells, like the induction of anti-apoptotic proteins of the cIAP family [141,142]. On the other hand, *H. pylori* LPS-stimulated MMP9 was shown to induce pro-apoptotic cleaved caspase 3 and reduced cell survival [140].

In addition to the observed anti-apoptotic effects and the stimulation of cell proliferation, proteases like ADAM17 and ADAM10 imprint a cancer stem cell phenotype in cells by shedding of Notch1, and thus support anchorage-independent growth [67]. Interestingly, a variant of *H. pylori* HtrA displaying a leucine residue at position 171 was also enriched in gastric cancer patients as compared to patients with non-ulcer dyspepsia and peptic ulcers. It can be speculated that a higher efficiency in basolateral CagA delivery by *H. pylori* is linked to a higher risk for developing gastric cancer [59].

## 5. Disease Progression in a Proteolytic Environment: Epithelial-Mesenchymal Transition, Metastasis, and Neo-Angiogenesis

Epithelial-mesenchymal transition (EMT) is a process in which epithelial cells lose the expression of epithelial traits and gain the expression of mesenchymal marker proteins. A characteristic of EMT is the loss of epithelial marker proteins (E-cadherin, catenins, etc.) expression either via transcriptional regulation, delocalization, or proteolytic degradation. In turn, the decreased E cadherin expression results in deregulated β-catenin signaling and the stabilization of the mesenchymal phenotype via transcription factors of the SNAIL, TWIST, and ZEB families [165]. *H. pylori* infection was suggested to induce an EMT-like phenotype in a number of studies (recently reviewed in [166]), and E-cadherin expression is frequently lost in premalignant metaplasia and early stages of gastric cancer [167], while only a smaller fraction of gastric cancer samples expressed the mesenchymal marker protein N-cadherin [168].

In fact, the proteolytic shedding of E-cadherin might contribute to the loss in epithelial stability and cell identity well before the transcriptional down-regulation during EMT [169]. The formation of soluble E-cadherin fragments shows a strong oncogenic potential, as these fragments can directly bind and activate receptor tyrosine kinases. Thereby, enhanced E-cadherin shedding is involved in induction of pro-oncogenic signaling pathways, such as the PI3K-Akt-mTor pathway or the MAPK-Erk pathway, and results in tumor cell growth, survival, and motility [68,170,171,172,173]. *H. pylori* infection also leads to the formation of two intracellular E-cadherin fragments, which are released into the cytosol [79]. Consequently, the intracellularly complexed β-catenin and p120-catenin are also released from the complex [174]. Translocation of β-catenin and p120-catenin have been observed in *H. pylori*-infected gastric epithelial cells as well. Once released from the E-cadherin complex, β-catenin binds the Tcf/lef transcription factors in the nucleus and enhances the transcriptional activity [175]. This effect is supported by nuclear p120-catenin binding the transcriptional repressor of Kaiso to relieve suppressed MMP7 expression [176].

EMT processes in gastric cancer are invariably associated with an inflammatory tissue microenvironment, and both factors synergize in unfolding the metastatic potential of transformed cells (Figure 2) [177]. Importantly, several proteases, which are frequently found in the gastritis- and gastric-cancer-associated tissue micromilieu, have been reported to foster EMT processes via several pathways. ADAM10 contributes the shedding of E-cadherin and c-Met [66]. Whilst E-cadherin cleavage clearly feeds into the mesenchymal transition [144], the loss of c-Met expression impairs HGF reactivity of the cells and thus reduces the malignant potential [178]. However, several studies suggest that *H. pylori* stimulates c-Met-associated pro-oncogenic signaling cascades [145,146]. In gastric cancer cell lines, EMT was critically dependent on ADAM17, and knockdown of ADAM17 was able to reverse EMT transition by abrogating signaling via the TGF-β/Smad axis [147]. Besides TGF-β signaling, the insulin-like growth factor (IGF) signal transduction cascade is crucially involved in EMT in gastric cancer [165,179], where MMP11 knockdown was able to diminish IGF1 signaling and concomitantly reduced the invasive potential of gastric cancer cells [160,161]. In vitro experiments using mammary epithelial cells showed that MMP3 exposure was sufficient to induce SNAIL-dependent EMT [72]. Further, MMP3 is an important effector molecule in the induction of WNT-induced β-catenin signaling during EMT [153]. Therefore, MMP3 has been suggested as a natural tumor-promoting factor [180]. In parallel to the disintegration of cell–cell junctions, cell–ECM interactions are also destabilized by proteases allowing invasive migration of transformed cells [72]. MMP7-dependent HB-EGF signaling was shown to reinforce EMT marker expression [51], and MMP7 inhibition decelerated migration and inhibited the invasive capacity of AGS cells [65]. The importance of ECM targeting enzymes for invasive growth and metastatic cell migration is highlighted by the fact that many of the matrix-targeting MMPs are critically involved in these processes [148]. The gelatinases MMP2 and -9 are crucial in invasion and metastasis and in several tumor entities [134], and the MMP9 promoter allele Rs3918242 is associated with a higher risk of metastasis in gastric cancer [158]. The migration of gastric tumor cell lines was also linked to MMP3, MMP9, and MMP10, which facilitated cell migration over a Matrigel layer, an in vitro model for invasiveness of transformed cells [38,154,155]. The membrane-type metalloproteinase MMP14 fosters migration and invasion in vitro, while in vivo MMP14 expression levels positively correlate with lymph node metastasis [163,164].

As pointed out previously, the tumor microenvironment in general, and in particular, the abundance of proteolytic enzymes is strongly influenced by other cell types, such as infiltrating immune cells or cancer-associated fibroblasts (CAFs). CAF-derived MMP3, secreted by tumor-associated myo-fibroblasts, is sufficient to promote AGS cell migration [152]. Similar observations have been made for MMP10 secreted by tumor-associated macrophages (TAMs) [159]. In a recent study, TAM-derived MMP9 was shown to support metastasis, and treatment with MMP9 inhibitors could reduce distant metastasis in gastric cancer [156]. Little is known about the direct involvement of proteases in angiogenic processes during gastric cancer progression. Remodeling of the ECM is a prerequisite for angiogenesis and self-evidently, MMPs are highly important in this step [181]. Additionally, MMP2 and MMP9 promote angiogenesis via stimulation of V-EGF [149,150,157] and activation of TGF-β signaling [151]. On the other hand, MMP12 activates angiostatin, which is a cleavage product of plasminogen and counteracts angiogenesis [162]. However, the association of MMP12 expression with higher survival rates in gastric cancer patients is controversial [37,182,183].

## 6. Concluding Remarks

*H. pylori*-driven gastric pathologies and gastric cancer are closely linked to inflammatory processes and proteolytic reshaping of the tissue microenvironment. The consequences of proteolytic enzymes derived from bacteria as well as host cells affect every level of the Correa cascade outlined above, and thus they can be considered significant contributors to gastric tumorigenesis. Proteases affect tissue architecture and ECM composition, they aid inflammatory processes and immune cell recruitment, and last but definitely not least, they directly target signaling cascades with well-established roles in cancer formation and progression. The variety of proteases involved and the diversity of protease substrates draws a complicated picture of interdependent disease mechanisms. The advent of organoid-based models and stomach-on-a-chip technologies might help to clarify the proteolytic contribution of the individual cells types present in the mucosal microenvironment of the stomach [139]. Nevertheless, targeting individual proteases could provide us with alternative strategies to fight *H. pylori*-associated disease and gastric cancer. In particular, proteases that contribute to late events in gastric cancer progression, such as metastasis and angiogenesis, might represent attractive targets for therapeutic intervention.

## Figures and Tables

**Table 1:** Proteases in colonization, epithelial inflammation, and epithelial barrier disruption.

Group/Protease	Putative Target	Importance
**Colonization and Mucosal Inflammation**		
ADAM17	proTNF-α [53], TGF-α [44]	pro-inflammatory [44,53] response transcriptional repression of the gastric H, K–adenosine triphosphatase α-subunit, reduced acid production [63]
MMP1 (collagenase-1)	proTNF-α, proIL-1β [42]	pro-inflammatory effect [64]
MMP2 (gelatinase A)	CXCL5 [46]	neutrophil recruitment [46], upregulated via IL-21 [48,49]
MMP7 (matrilysin-1)	proTNF-α [65] HB-EGF [51,54]	reduced inflammation, dampens the production of the cytokines [22], immune cells infiltration [24]
MMP9 (gelatinase B)	CXCL5 [46]	neutrophil recruitment [45,46], upregulated via IL-21 [48,49], macrophages infiltration [56], pro-inflammatory and anti-inflammatory activity [64]
MMP10 (stromelysin-2)		supports bacterial colonization, fosters tissue inflammation, recruitment of T cells [23]
Hp0169	Type I collagen	colonization of Mongolian gerbils [21]
HtrA		chronic inflammation [59]
**Impairment of Junctional Integrity and Epithelial Barrier Function**		
ADAM10	E-cadherin [66]	loss of AJ [66], stem-like phenotype in cancer stem cells and supports anchorage independent growth [67]
ADAM15	E-cadherin [68] desmoglein-2 [69]	impaired cell adhesiveness [69]
ADAM17	desmoglein-2 [69]	impaired cell adhesiveness [69], stem-like phenotype in cancer stem cells and supports anchorage independent growth [67]
MMP2 (gelatinase A)	Occludin [70]	increase in epithelial or endothelial permeability [70]
MMP3 (stromelysin-1)	E-cadherin [71]	disintegration of cell-cell junctions, destabilization of cell-ECM interactions [72]
MMP7 (matrilysin-1)	E-cadherin [51,71,73]	abnormal cell aggregation and increase in cells invasiveness [64]
MMP9 (gelatinase B)	Occluding [74] E-cadherin [75] desmoglein-2 [76]	impaired cell adhesiveness [76]
MMP14 (MT1-MMP)	E-cadherin [77]	adhesion reduction [64]
Hp1012	JAM-A [78]	impaired barrier function, reduced intercellular adhesion and increased invasive potential of epithelial cells [78]
HtrA	E-cadherin [79,80] desmoglein-2 [80] occludin, claudin [81]	disruption of intercellular junction access to basolateral space [79,80,81]
caspase-3	E-cadherin [82,83] desmoglein-2 [84]	
calpain	E-cadherin [85] desmoglein-2 [86]	

**Table 2:** Proteases in EMT, tissue transformation, metastasis, and neoangiogenesis.

Group/Protease	Putative Target	Importance
**Neoplastic Transformation, Proliferation, and Cell Survival**		
ADAM9 and -15		gastric cancer cell lines proliferation [117]
ADAM10	Notch1 [67]	stem-like phenotype in cancer stem cells and supports anchorage independent growth [67]
ADAM12		decreases gastric cancer cell lines proliferation [117]
ADAM17	HB-EGF [53] Notch1 [67]	reduces apoptosis [135], poor prognosis in gastric cancer [133], induces pro-survival signaling via the EGFR [44,135,136], cancer stem like phenotype, anchorage-independent growth [67]
MMP7 (matrilysin-1)		promotes proliferation in non-transformed epithelial cells [132]
MMP12 (macrophage metalloelastase)		inversely correlates with disease outcome [37,134]
caspase-3		executioner caspase, activation by caspase-8, -9, or -10 [139], activation induced by *H. pylori* LPS and induced MMP9 [140], *H. pylori* induction of anti-apoptotic proteins of the cIAP family to reduce caspase-3-dependent apoptosis [141,142]
caspase-8		initiator caspase, limited proteolytic (including autocatalytic) activity, engaged by death receptors, including tumor necrosis factor receptor 1 (TNFR1) and Fas/CD95 [139], TieA-protein-induced apoptosis [143]
HtrA		HtrA L171 variant was enriched in gastric cancer patients and may increase efficiency in basolateral CagA delivery by *H pylori* and risk for developing gastric cancer [59]
**Epithelial-Mesenchymal Transition (EMT), Metastasis and Neo-Angiogenesis**		
ADAM10	E-cadherin [66,110] c-Met [66]	E-cadherin cleavage induced EMT [144], c-Met-associated pro-oncogenic signaling cascades [145,146]
ADAM17		EMT [147]
MMP2 (gelatinase A)		invasive growth, angiogenesis [134,148,149,150,151], cell migration [152], cell proliferation, migration of epithelial cells [64]
MMP3 (stromelysin-1)		SNAIL-dependent EMT [72], WNT-induced β-catenin signaling [153], migration [152,154], invasion [154], angiostatin-like fragments, cell proliferation, release of VEGF, upregulation of angiogenesis [64]
MMP7 (matrilysin-1)		EMT marker expression [51], migration and invasion capacity [65], proliferation [132], cell differentiation, vasoconstriction and cell growth [64]
MMP9 (gelatinase B)		invasive growth [134,148,155,156], angiogenesis [134,148,149,151,157], tumor cell resistance [64], promoter allele Rs3918242 is associated with metastasis [158], tumor-associated macrophages-derived MMP9 supports metastasis in gastric cancer [156]
MMP10 (stromelysin-2)		invasion [38], migration [159], tumor-associated macrophages-derived MMP10 promotes cell migration [159]
MMP11 (stromelysin-3)		IGF1-dependent invasive potential of gastric cancer cells [160,161], decreases cancer cell sensitivity to NK cells [64]
MMP12 (macrophage metalloelastase)		angiogenesis [134,162]
MMP14 (MT1-MMP)		cell migration, epithelial cell migration, adhesion reduction [64], migration and invasion [163,164]

## Data Availability

Not applicable.
